# A Survey of People With Parkinson’s and Their Carers: The Management of Pain in Parkinson’s

**DOI:** 10.1177/08919887211023592

**Published:** 2021-07-08

**Authors:** Jenni Naisby, Anneesa Amjad, Natasha Ratcliffe, Alison J. Yarnall, Lynn Rochester, Richard Walker, Katherine Baker

**Affiliations:** 1 Department of Sport, Exercise and Rehabilitation, Northumbria University, Newcastle Upon Tyne, United Kingdom; 2 Clinical Ageing Research Unit, Institute of Neuroscience/Newcastle University Institute for Ageing, Newcastle University, Newcastle Upon Tyne, United Kingdom; 3 MS Society, London, United Kingdom; 4 Parkinson’s UK, London, United Kingdom; 5 Northumbria Healthcare NHS Foundation Trust, North Tyneside General Hospital, North Shields, United Kingdom

**Keywords:** Parkinson’s disease, patient and public involvement

## Abstract

**Background:**

Pain in Parkinson’s is problematic but under treated in clinical practice. Healthcare professionals must understand the impact of pain in Parkinson’s and patient preferences for management.

**Objective:**

To understand the impact of pain in Parkinson’s and to understand current management and preferences for pain management.

**Methods:**

We conducted a national survey with 115 people with Parkinson’s (PwP) and 10 carers. Both closed and open questions were used. The questions focused on how pain affected the individual, healthcare professional involvement in supporting pain management, current pain management strategies and views on future pain management interventions. We used descriptive statistics to summarize closed responses and thematic analysis to summarize open question responses.

**Results:**

70% of participants reported pain impacted their daily life. Pain had a multifactorial impact on participants, affecting movement, mood and quality of life. Improved pain management was viewed to have the potential to address each of these challenges. Pain affected a number of different sites, with low back pain and multiple sites being most frequently reported. Exercise was the most frequently noted strategy (38%) recommended by healthcare professionals for pain management. PwP would value involvement from healthcare professionals for future pain management, but also would like to self-manage the condition. Medication was not suggested as a first line strategy.

**Conclusions:**

Despite reporting engagement in some strategies to manage pain, pain still has a wide-ranging impact on the daily life of PwP. Results from this survey highlight the need to better support PwP to manage the impact of pain.

Pain can affect up to 85% of people with Parkinson’s (PwP)^1,2^ yet is poorly recognized and managed within clinical practice,^3^ which can have significant consequences on quality of life.^4^ Pain is biopsychosocial and has a multifactorial impact on individuals lives, yet this has been found to not be acknowledged by healthcare professionals.^5^ Despite the problem of pain, there are limited options for pain management in Parkinson’s.^6^ Between 50%^7^ and 63%^8^ of those reporting pain have not received any pharmacological or non-pharmacological treatment for their pain, which has been attributed to an inadequate awareness of clinicians.^9^ There is a need to increase education concerning pain in Parkinson’s for healthcare professionals and to develop options for both pharmacological and non-pharmacological management of pain.^10^ Currently, it is unknown if pharmacological therapy will relieve pain in Parkinson's due to the distinct mechanisms involved with pain processing interacting with Parkinson’s pathophysiology.^11^ A small number of cross sectional studies have provided some detail on how PwP manage their pain. Physiotherapy and pain medication are the most frequently cited approaches.^7,8,12^ However, this must be considered in the wider context of a number of individuals not receiving any treatment. There is limited literature to date exploring the impact of pain, current management strategies and the preferences of PwP for future interventions. Understanding of these factors will help to inform the development of pain management for PwP.

Currently there is only a small amount of research that focuses specifically on PwP views on pain. This work aimed to enable PwP and their carers to share their experiences of pain and pain management in their own words and, importantly, to share their thoughts on what pain management interventions should look like. This survey forms part of a wider piece of patient and public involvement to inform the direction of a future research study about pain management in Parkinson’s.

## Materials and Methods

### Ethics

The survey was approved by Northumbria University Research Ethics committee.

Informed consent was obtained at the start of the survey. We confirm that we have read the Journal’s position on issues involved in ethical publication and affirm that this work is consistent with those guidelines.

### Developing the Survey

In order to ensure the survey was clear and that questions focused on aspects important to PwP, we considered it vital to work with PwP to develop the survey questions. JN, AA and NR drafted questions based on existing literature and the research team’s plans for a future study. The draft survey was then sent to 5 people affected by Parkinson’s (3 male & 2 female; time since diagnosis: ranging between 2-15 years) who provided feedback on the clarity of questions and recommended changes. As a result of this feedback, a number of amendments were made including reducing the length of the survey, refining the focus and providing more detail for certain questions.

### Recruitment and Procedures

The survey was administered in March 2020 by Parkinson’s UK on SmartSurvey, an online survey software and questionnaire tool. Participants were recruited via Parkinson’s UK Research Support Network—an online network that brings together people driven to help find a cure and better treatments for Parkinson’s. The Research Support Network has around 6,000 members (as of February 2020), the vast majority of whom are PwP and partners, family members and carers of those with the condition living in the UK. An email was sent to the network inviting people to complete the survey, and the survey was also included in the Network’s monthly e-newsletter.

The survey gathered feedback from both PwP and partners, family members and carers. The target population was those who had experience of pain. The question set was the same for the 2 groups, aside from minor variations in wording to make questions applicable. The first question required the individual to indicate if they were a person with Parkinson’s or a partner, family member or carer so the appropriate question set could be shown (supplementary material). The survey consisted of 2 parts. Part 1 included 8 questions focused on how pain affected the individual (or their partner/family member/person they cared for), their experience of healthcare professional involvement in supporting pain management, current pain management strategies and views on future pain management interventions. Three questions collecting demographic information were also included. Part 2 consisted of a further 9 questions focused on feedback for the specific design of a study. Results from part 2 are not reported here. The survey did not collect any identifying information. Participants were able to skip any questions they did not wish to answer. The survey included a mixture of closed and open-ended questions. Open ended questions were deemed important to allow participants to develop responses due to the limited research in this area, and also provided an opportunity for participants to highlight any additional points they deemed relevant.

### Data Analysis

We used descriptive statistics to characterize the sample. Categorical data were analyzed via SPSS (version 21) to generate frequencies. Open ended questions were summarized using thematic coding analysis.^12^ These were informed by the aims of the research, while allowing new themes to emerge. One author (JN) read through the responses, coded these and then categorized into themes. A second author (KB) independently categorized the coded responses and a final presentation of themes was agreed through a peer debriefing meeting between the 2 authors and discussed with the wider team.

## Results

We received responses from 115 PwP and 10 carers. Almost all the questions (except 3) were focused on the experience of the person with Parkinson’s, with partners, family members and carers responding on the person’s behalf. Therefore, the reporting of “participants” refers to the information captured from both groups of respondents (N = 125), unless specified otherwise. All participants answered the closed questions (see Table 1), and the number of responses to the open questions is indicated within the Tables 2-5. Participants could choose more than 1 answer to the closed and open questions.

**Table 1.** Participant Demographics and Diagnosed Questions Responses.

Demographic item from survey	Responses
Age	
Under 30	0
30-49	2 (2%)
50-59	30 (24%)
60-69	41 (33%)
70-79	42 (34%)
80 or over	10 (8%)
When diagnosed with Parkinson’s	
< 2 years ago	19 (15%)
2-5 years ago	53 (42%)
6-10 years ago	35 (28%)
11-20 years ago	17 (14%)
More than 20 years ago	1 (1%)
Does pain impact your daily life?	
Yes	88 (70%)
No	28 (22%)
Not sure	9 (7%)
How frequently do you experience pain?	
a. Never	9 (7%)
b. Rarely (around once per year)	7 (6%)
c. Sometimes (around once per month)	18 (14%)
d. Frequently (around once per week)	61 (49%)
e. Very frequently (most days)	30 (24%)
Is pain a symptom you discuss with your healthcare professional?	
a. No I haven’t discussed pain with a healthcare professional	37 (30%)
b. Yes I’ve discussed with my GP (General Practitioner)	48 (38%)
c. Yes I’ve discussed with my Parkinson’s nurse	60 (48%)
d. Yes I’ve discussed with my Physiotherapist	44 (35%)
e. Yes I’ve discussed with my Occupational Therapist	9 (7%)
f. Yes I’ve discussed with my Dietician	1 (0.8%)
g. Yes I’ve discussed with another healthcare professional (please detail)	33 (28%)
Consultant	14 (11%)
Neurologist	12 (10%)
Pain clinic	3 (2%)
Chiropractor	3 (2%)
Gastroenterologist	1 (0.8%)
What did the healthcare professional do to support you with your pain?	
a. Nothing	12 (10%)
b. Advice, education or information	36 (30%)
c. Medication	29 (25%)
d. Exercise	44 (38%)
e. Complementary therapy (e.g. massage, acupuncture)	19 (17%)
f. Other (please detail)	18 (16%)
Injections	1 (0.8%)
Botox	1 (0.8%)
Psychologist referral	1 (0.8%)
Physiotherapy referral	3 (3%)
Consultant referral	3 (3%)
Pain not related to Parkinson’s	2 (2%)
Aid	1 (0.8%)
Leaflet	1 (0.8%)

**Table 2.** Management Strategies for Pain.

Category	Sub category	N	Example
Physical activity	Walking	9	Walking and trying to keep fit
	Pilates	8	If I arrive at Pilates feeling bad, I leave feeling better
	Gym	5	I try to go to the gym
	Swimming/ hydrotherapy	3	I swim when I can
	Stretching	9	Do stretching exercises
	Exercise	35	Lots of exercise
	Yoga	6	Yoga to stretch my muscles
	Dance	3	PD dancing
	Exercise class	9	Parkinson’s exercise class
	Cycling	1	Occasional cycle
Medication	Paracetamol	19	Paracetamol 8 daily
	Ibuprofen	14	Taking lots of ibuprofen every day
	Co-codamol	2	Taking co-codamol 500mg 2 x day
	“Painkillers”	10	I take painkillers
	Pregabalin	2	Pregabalin medication 70 mg twice daily
	Gabapentin	2	Initially Gabapentin, then pregabalin, pain clinic but medication stopped working. They have not offered anything else.
	Amitriptyline	1	10mg amitriptyline daily
	Fluoxetine	1	I take fluoxetine, which really helps with the stiffness and some of the pain
	Aspirin	1	Aspirin
	Codeine	1	Codeine
	Sinemet	2	Also related to wearing off Sinemet, so next dose may relieve things
	Zapain	1	Zapain capsules up to 8 in 24 hour period
	Hesitation taking medication	5	Have not tried Gabapentin…concerns about sedation
Other therapies	Massage/ manual therapy	10	Occasional massage
	Mindfulness	5	I have tried…Bowen technique…mindfulness
	Bowden technique	2	Mental relaxation e.g. reflexology
	Reflexology	3	Also undergone reflexology, acupuncture,…mindfulness
	Acupuncture	1	I also use cannabis oil
	TENS	1	
	Cannabis oil	2	
Support from healthcare professionals	Physiotherapy	9	Regular physiotherapy
	Chiropractor	2	Have consulted with osteopaths, chiropractors
	Osteopath	1	Pain clinic (1 visit)
	Pain clinic	1	
Multiple strategies	Medication and exercise	15	Exercise, paracetamol
	Medication, exercise and other therapies	4	Regular exercise…mental relaxation e.g. reflexology, massage, very occasionally ibuprofen
	Medication, healthcare and exercise	4	Private physiotherapy, directed exercise, ibuprofen
	Exercise and other therapies	3	I exercise and I stretch…I often for a massage within financial constraints
	Healthcare, other therapies and exercise	4	I do what I can by exercise…do some sort of exercise with a neuro physiotherapist…then if I had worked hard enough I would get a sports massage
No management options have been identified or successful	Tried a range of strategies	3	I have had massage and do regular Pilates and stretches, with not a lot of benefit
	No/unsure	9	Not sure what to do
	Nothing helps	5	
No qualitative response provided		14	

**Table 3.** Pain Impact on Day to Day Life.

Category	Sub category	N	Example
Type and site of pain	Experience multiple symptoms	24	Long term frozen shoulder, joint and muscle pain, tendonitis
	Low back pain	32	Back is painful, probably due to posture changes
	Shoulder pain	18	Sharp pain and ache in right shoulder
	Joint pain	5	Joint pain
	Muscle pain	3	My muscles contract
	Nerve pain	3	Neurological pain is felt in my toes
	Cramps/spasms	5	Dystonia
	Tendinitis	1	
	Arthritis	3	Have arthritis in my back, not sure if the pain is this or PD
	Hip pain	5	It is permanently in the hip which radiates down the front
	Facial pain	1	Pain from tremor
	Pain linked to tremor	3	It moves and changes
	No pattern of pain	4	Abdominal pain, cant get out of bed
	Internal pain	3	
	Headache	1	
	Wearing off pain	4	Frequent back ache when wearing off
	Restless legs	1	Front of my leg from knee to ankle to top of my foot
	Lower limb pain	12	
	Stiffness	10	No pain just stiffness
	Night pain	5	It wakes me up at night
	Morning pain	5	On getting up in the morning, difficulty in moving
Biopsychosocial impact	Impacts movement	18	It makes it difficult to walk
	Pain impacts mood	5	I do get the impression healthcare professionals do not realize the extent of the pain which feedbacks into my low mood/anxiety
			Sometimes I feel overwhelmed by pain
	Feeling overwhelmed	1	It interrupts my sleep and makes me feel pretty miserable most of the time
	Impacts sleep	2	It makes me miserable and at times frightened
	Feels frightened	2	When the pain and tightening occur they are often in association with weakness
	Impacts strength	2	It affects me probably 75-80% of my week
	Impacts daily life	7	Daily pain was 1 reason I retired early
	Influenced retirement	1	
No qualitative response		34	

**Table 4.** Expectations From a Pain Management Intervention.^a^

Category	Sub category	N	Example
To reduce pain	To be pain free	1	To be pain free for longer periods of time
	To provide pain relief	42	Reduction of the pains
	Impact mood	2	My pain makes my mood worse
To find the cause of pain	Diagnosis	8	To find out what is causing the pain
	MRI scan	1	MRI as appropriate
Nature of the intervention	Healthcare professional supported	17	I would like to speak with professionals who have an interest in this area—explain my challenges—together see what is possible
	Self-manage	5	Would love something I can manage myself, then I don’t have to go to appointments
	Healthcare professional support and self-management	6	Advice and guidance from a professional while getting on with it one-self
	Exercise		Exercises would be best
	Non drug approach	9	Non drug related relief
	To improve function	5	A reduction in pain would allow me to function better
	A holistic intervention	1	Fully holistic approach
Don’t know		8	No idea
No qualitative response		17	

^a^ Response to questions: “What would you expect from a pain management intervention?” “Would you want a healthcare professional to be involved, or would you prefer something that could self-manage?”

**Table 5.** Expected Impact of a Better Pain Management Strategy?^a^

Category	Sub category	N	Example
Improve wellbeing	Change life	9	It would change my life
	Improve activity	18	Make me more active
	Improve mood	9	Feel better mood wise
	Improve sleep	8	Improved sleep duration and pattern
	Improve QoL	11	A much better quality of life
	Impact hobbies	3	Freedom to enjoy daily activities
	Impact social life	2	I could socialize without having to worry I’ll have to return home
	Impact job	1	I would be able to do my job without pain
	Impact daily life	7	Make my life better
	Mental and physical improvement	3	A mental and physical impact
	Decrease symptom burden	2	It would change the outcome of everyday life
To impact management	Reassurance	3	Reassurance that the issue is being addressed
	Control	8	A feeling of being in control
	Knowledge	3	Feel I would be getting all the advice I need
Don’t know		5	Not sure, I will try anything
No qualitative response		17	

^a^ NB: In response to “What impact would a better pain management strategy have?”

### Closed Questions

Please see Table 1 for details of participant characteristics. 75% of participants were aged 60 or over. 42% had been diagnosed between 2 and 5 years. A large majority of participants indicated pain impacted their daily life (70%), with 49% experiencing pain multiple times per week and 24% experiencing pain multiple times per day. The healthcare professional (HCP) with whom the highest number of participants had discussed their pain was their Parkinson’s nurse (48%) followed by a general practitioner (38%) and a physiotherapist (35%). 10% of PwP discussed pain with their neurologist. Exercise was the most frequently noted mechanism by which healthcare professionals supported individuals with their pain (38%).

### Open Questions

The open-ended questions provide detailed information regarding the current impact of pain, it’s management and future management suggestions.

#### The impact and current management of pain in Parkinson’s

When asked about what pain management strategies PwP used, physical activity, paracetamol and ibuprofen were the most frequently reported. Some individuals described multiple strategies, with some participants identifying a wide range of strategies that had been tried to manage their pain. Other participants acknowledged that no management options had been successful to date or not being sure how to manage their pain. While medication was cited, some participants expressed reluctance at taking this. Parkinson’s medication was not linked with pain. Five individuals noted painful muscle spasms and cramps. Some participants had tried other therapies such as massage and mindfulness. Table 2 provides a description of each of the pain management strategies and examples identified by participants. When asked if pain impacted their daily life, responses focused around describing the location of their pain and the timing of this, alongside capturing the biopsychosocial impact that this can have. Pain was reported in a number of locations, with low back pain being the most frequently reported, alongside having pain in multiple locations. Wearing off pain was noted by few participants. Pain made movement difficult for some participants, alongside having a negative impact on mood and wider daily life. Detail surrounding these open-ended responses is provided in Table 3.

#### Pain management needs for people with Parkinson’s

Participants identified the need for pain management interventions to provide pain relief. Involving healthcare professionals with pain management was deemed important, with some individuals viewing it necessary to be able to self-manage their pain or to have a combination of support and self-management. When asked an open question about future pain management medication was not mentioned as a suggestion for pain management, however some participants highlighted a non-drug approach to be the preference. Table 4 provides further detail pertaining to the nature of pain management interventions suggested by participants. Participants indicated improved pain management would provide an overall positive impact on general wellbeing, in particular improving activity and quality of life. An impact on mood, sleep and daily life were often referred to alongside improved pain management as having the potential to “change the life” of some individuals. A sense of being in control of pain was deemed important by some. Table 5 elaborates on these findings, with specific examples provided.

## Discussion

This survey has identified the anticipated impact improved pain management strategies would have on PwP living with pain, alongside preferences on how such strategies should be delivered. Studies to date present findings on strategies that have been tried for PwP but have involved little dialogue with people about their preferences. This survey provides a direct account of what PwP would like from future pain management interventions and the potential impact they felt that this would have.

Pain was highlighted as having a biopsychosocial impact, with movement, mood and daily life each being cited as being impacted by pain. Despair and despondency associated with living with pain in Parkinson’s has been reported in a qualitative study (n = 4).^5^ This study reported individuals experiencing high levels of pain which had psychological and social impacts. Some participants tried management strategies such as exercise, while others felt this to not be possible. Having a sense of control over pain was key. A disconnect between healthcare professionals and PwP was highlighted, with individuals needs surrounding pain not addressed.^5^ While small samples can be expected in this type of study, transferability of these findings is limited as a stand-alone study. The current survey adds to these findings, highlighting on a larger scale the wide-ranging impact of pain, and variation among the use of strategies. A key development with the current survey is individuals reporting how improved pain management would impact their daily life. A feeling of overall improved wellbeing including improved activity, mood and quality of life were each highlighted if pain were to be better managed. Pain can influence all aspects of someone’s life^13^ and be a particularly bothersome symptom.^14^ Individuals in this survey noted the dominating effect of pain, and the overarching positive impact that a reduction in pain could have on daily life.

Exercise is encouraged for PwP as a key aspect of the management of the condition.^3,15^ The current survey identified that exercise is a frequently advised strategy by healthcare professionals to support the management of pain. At early stages of Parkinson’s it has been found that activity levels are reduced compared to age matched controls.^16^ Pain can be a barrier to exercise^15^ and increased physical activity has been found to be associated with worse pain scores in Parkinson’s.^17^ Within the literature related to chronic musculoskeletal pain, avoidance of activity due to fear and reduced self-efficacy regarding managing symptoms can occur.^18^ Keeping active may not seem plausible to patients with low perceived control over their pain^19^ and has been identified as an issue with PwP and pain.^13^ While exercise is also advocated for the management of pain in the general population^20,21^ this is often in conjunction with other strategies including education and support.^22,23^ Given that PwP in this survey highlighted exercise as a key strategy for reducing pain, future work to investigate pain management strategies specific to Parkinson’s is necessary.

Medication was also identified as a method currently used to manage pain. The most frequently cited medication was paracetamol (15% of individuals) followed by ibuprofen (11% of individuals), which follows a similar pattern to cross sectional studies, which have collected medication use data.^2,12^ This type of medication use has been criticized as not targeting the central mechanisms of pain in Parkinson’s.^2^ Alongside this, these are not large numbers of the participants reporting medication use, and often individuals do not receive any support with pain management, including medication.^7,8^ Some participants in the current survey identified a hesitance at taking medication or suggesting a non-drug approach for future management. It is important to note that in an open question about expectations for a pain management intervention, medication was not suggested as a potential strategy by participants.

Healthcare professional support was valued by participants for pain management, appreciating the knowledge and advice healthcare professionals could provide being most frequently cited. A recent systematic review of pain management for PwP^6^ advocates the need for further research in this area. The review demonstrated promise for pharmacological therapy focusing on Safinamide, and from 1 study, efficacy for multidisciplinary team (MDT) management. “Miscellaneous therapies” were highlighted including hydrotherapy, massage and resistance exercise, yet the quality of these studies was poor. Pain is multifactorial,^24^ and individual approaches like those approaches did not reflect the biopsychosocial impact of pain. The review was comprehensive in its search, including allied health, behavioral therapy and client centered therapy, yet identified little in these areas. While MDT management had shown promise for pain^25^ this was not the primary outcome or focus of this study and there was limited detail of the MDT management. A previous cross sectional survey found the greatest efficacy for pain management was reported in conjunction with treatment in a rehabilitation clinic or physiotherapy.^12^ To date, there is limited literature focusing on interventions for pain management in Parkinson’s.^6^ Pain management in the context of wider care, with support from healthcare professionals warrants investigation given PwP identifying the wide ranging impact of pain on their daily lives and a preference for support in pain management.

The participants in this study identified a number of different HCPs with whom they have discussed their pain with. In previous studies an orthopaedic doctor or general practitioner were the most frequently cited, with a small number citing neurologists.^12^ In agreement, the current survey found a small number of neurologists and consultants cited. However, the Parkinson’s nurse and physiotherapist were frequently referred to as those who individuals had consulted. This finding is crucial as it indicates that there is a need for a multidisciplinary approach to pain management in Parkinson’s, in support of previous work.^25^ HCPs working with individuals with chronic musculoskeletal pain have reported challenges in supporting people with pain and require support themselves.^26^ Alongside this, literature exploring clinician attitudes and beliefs to chronic musculoskeletal pain has found HCP attitudes and beliefs to be associated with those of their patient. A biomedical orientation has a negative association with patient education, adherence to treatment guideline and activity recommendations.^27^ These results of the current survey show that PwP feel HCPs have a key role to play in pain management. It is therefore important for future work to understand attitudes and beliefs among HCPs regarding pain in Parkinson’s, particularly given the limited focus on pain management in current Parkinson’s practice.^7,8^

70% of participants reported pain influenced their daily life, which aligns with other cross sectional studies focusing on pain and Parkinson’s, which report between 52%^28^ and 85%^2^ of people reporting pain. The current survey found 49% to experience pain daily and 24% multiple times per day. These findings are higher than previously reported of up to 21% “often” experiencing aches and pains and 6% “always” experiencing these.^29^ Low back pain is frequently cited as problematic in Parkinson’s^30,31^ and was noted most frequently in this survey, followed by individuals experiencing pain at multiple sites. A diagnosis of the cause of pain was important to some individuals. However, given the central processes involved in pain, and PwP potentially being more predisposed to pain^32,33^ a definitive diagnosis may be difficult to achieve. A diagnosis can provide legitimacy to pain, and one potential way to achieve this is to consider education regarding pain physiology.^18^ This captures the biopsychosocial nature of pain, supporting participants to understand the multifactorial influences on a pain experience. The participants focused predominantly on the location of pain within this survey. Given that there are often different pain mechanisms involved with pain and Parkinson’s^34^ communicating these to PwP may help with understanding.

A limitation of this study is the lack of measures of disease severity including Hoen and Yahr staging, MDS UPDRS and measures of non-motor symptoms such as sleep and mood. This was a non-random sample, using self-report data, which is also acknowledged as a limitation of the study. Those responding are more likely to have had a problem with pain in view of the nature of the study. Alongside this, as individuals recruited would have expressed an interest to be contacted regarding research, the population may not be entirely representative of the Parkinson’s population as a whole. The relationship between pain, OFF periods and anti Parkinson medication was not specifically explored, however pain has been found to be no different between the ON and OFF state in the largest pain and Parkinson’s study to date.^2^ We did not use a validated scale to capture information regarding pain in Parkinson’s, for example the Kings Parkinson’s pain scale.^35^ However, the survey was able to address the aim of this exploratory study, to identify current pain management strategies and suggestions for the future. There were responses from 10 carers within this survey and it should be acknowledged they may have differing views to the PwP themselves, though there were not sufficient data to investigate this here. Respondents were all from the United Kingdom, which has implications for the availability of services.

The key strength of this study is that it has explored preferences and needs regarding pain management by directly asking PwP for their views. Feedback from a small group of PwP on the draft survey questions helped ensure that the survey focused on aspects important to PwP, and the use of open-ended questions allowed PwP to make suggestions about what pain intervention strategies should look like. Key HCPs who could be involved in pain management have also been identified, which will help when providing tailored support and guidance to healthcare professionals themselves. This study highlights the need to develop evidence surrounding how to manage pain in Parkinson’s, alongside the potential impact improved management would have on PwP quality of life, movement and wellbeing.

## Supplemental Material

Supplemental Material, sj-pdf-1-jgp-10.1177_08919887211023592 - A Survey of People With Parkinson’s and Their Carers: The Management of Pain in Parkinson’s Click here for additional data file. Supplemental Material, sj-pdf-1-jgp-10.1177_08919887211023592 for A Survey of People With Parkinson’s and Their Carers: The Management of Pain in Parkinson’s by Jenni Naisby, Anneesa Amjad, Natasha Ratcliffe, Alison J. Yarnall, Lynn Rochester, Richard Walker and Katherine Baker in Journal of Geriatric Psychiatry and Neurology
