# Supplementary material for: A Survey of People With Parkinson’s and Their Carers: The Management of Pain in Parkinson’s
Source: J Geriatr Psychiatry Neurol. 2021 Jul 8;35(4):613–21. doi: 10.1177/08919887211023592 (PMC9210119; doi:10.1177/08919887211023592)
Supplement: Supplemental Material, sj-pdf-1-jgp-10.1177_08919887211023592 - A Survey of People With Parkinson’s and Their Carers: The Management of Pain in Parkinson’s [file sj-pdf-1-jgp-10.1177_08919887211023592.pdf]

Supplementary materials

Survey

What is your association with Parkinson's?
___ I have Parkinson's
___ I am a carer/partner/family member or friend

People with Parkinson's

- What age are you?
    a. ___ Under 30
    b. ___ 30-49
    c. ___ 50-59
    d. ___ 60-69
    e. ___ 70-79
    f. ___ 80 or over

- When were you diagnosed with Parkinson's?
    a. ___ Less than 2 years ago
    b. ___ 2-5 years ago
    c. ___ 6-10 years ago
    d. ___ 11-20 years ago
    e. ___ More than 20 years ago

- Does pain impact your day-to-day life?
    a. Yes
    b. No
    c. Not sure

  Please tell us how your pain affects you:

- How frequently do you experience pain?
    a. Never
    b. Rarely (around once per year)
    c. Sometimes (around once per month)
    d. Frequently (around once per week)
    e. Very frequently (most days)

- Is pain a symptom you discuss with your healthcare professional?
    a. No I haven't discussed pain with a healthcare professional
    b. Yes I've discussed with my GP (General Practitioner)
    c. Yes I've discussed with my Parkinson's nurse
    d. Yes I've discussed with my Physiotherapist
    e. Yes I've discussed with my Occupational Therapist
    f. Yes I've discussed with my Dietician
    g. Yes I've discussed with another healthcare professional (please detail)

- What did the healthcare professional do to support you with your pain?
    a. Nothing
    b. Advice, education or information
    c. Medication
    d. Exercise
    e. Complementary therapy (e.g. massage, acupuncture)
    f. Other (please detail)

- Are you doing anything to help manage your pain?

- What would you expect from a pain management intervention?

- Would you want a healthcare professional to be involved, or would you prefer something that could self-manage?

- What impact would a better pain management strategy have on you?

Carers

- What age is the person with Parkinson's?
    a. ___ Under 30
    b. ___ 30-49
    c. ___ 50-59
    d. ___ 60-69
    e. ___ 70-79
    f. ___ 80 or over

- When were they diagnosed with Parkinson's?
    a. ___ Less than 2 years ago
    b. ___ 2-5 years ago
    c. ___ 6-10 years ago
    d. ___ 11-20 years ago
    e. ___ More than 20 years ago

- Does pain impact their day-to-day life?
    a. Yes
    b. No
    c. Not sure

Please tell us how your pain affects them:

- How frequently do they experience pain?
    a. Never
    b. Rarely (around once per year)
    c. Sometimes (around once per month)
    d. Frequently (around once per week)
    e. Very frequently (most days)

- Is pain a symptom they discuss with their healthcare professional?
    a. No they haven't discussed pain with a healthcare professional
    b. Yes they've discussed with their GP (General Practitioner)
    c. Yes they've discussed with their Parkinson's nurse
    d. Yes they've discussed with their Physiotherapist
    e. Yes they've discussed with their Occupational Therapist
    f. Yes they've discussed with their Dietician
    g. Yes they've discussed with another healthcare professional (please detail)

- What did the healthcare professional do to support them with their pain?
    a. Nothing
    b. Advice, education or information
    c. Medication
    d. Exercise
    e. Complementary therapy (e.g. massage, acupuncture)
    f. Other (please detail)

- Is the person with Parkinson's doing anything to manage their pain?

- What would you expect from a pain management intervention?

- Would you want a healthcare professional to be involved, or would you prefer something that the person with Parkinson's could manage themselves/with your support?

- What impact do you think a better pain management strategy would have on them?
